# Persistent Delirium in the Elderly: A Case Report and Review of Multifactorial Contributors and Management Challenges

**DOI:** 10.1155/crps/9751488

**Published:** 2026-06-25

**Authors:** Troy Salisbury

**Affiliations:** ^1^ Department of Psychiatry, Loma Linda University, Loma Linda, California, USA, llu.edu

**Keywords:** alcohol use disorder, geriatric psychiatry, hyponatremia, mirtazapine, persistent delirium

## Abstract

**Introduction:**

This case describes persistent delirium exceeding 18 months with prominent paranoid delusions in a 72‐year‐old woman with chronic hyponatremia, poststroke epilepsy, and alcohol use disorder. It highlights diagnostic and pharmacologic management challenges when standard agents carry prohibitive risks.

**Patient’s Main Concerns and Important Clinical Findings:**

A 72‐year‐old woman with a history of left hemispheric hemorrhagic stroke, poststroke epilepsy, and chronic alcohol use presented with acute confusion. Laboratory evaluation revealed severe hyponatremia (Na 122 mmol/L). Clinical findings included persecutory delusions, severe agitation with dangerous behaviors (attempting to leave moving vehicles, stabbing attempt), anxiety, insomnia, and fluctuating cognitive status with return to baseline between episodes. EEG demonstrated epileptiform discharges and generalized encephalopathy. Divalproex sodium levels at presentation were supratherapeutic (up to 127 mcg/mL). There was no evidence of prior dementia or progressive cognitive decline.

**Primary Diagnoses, Interventions, and Outcomes:**

The primary diagnosis was persistent delirium with multifactorial etiology. Interventions included hyponatremia correction, transition from divalproex to lacosamide, and initiation of mirtazapine, which was selected for its lower hyponatremia risk compared to other antidepressants, lower seizure risk compared to antipsychotics, and low deliriogenic potential. Sleep and anxiety improved with partial agitation reduction, though delirium persisted. Over 18 months, paranoid delusions evolved from episodic to continuous, creating treatment adherence barriers.

**Conclusion:**

This case underscores the potential for exceptionally prolonged delirium in medically complex older adults, diagnostic complexity in differentiating persistent delirium from ictal‐related psychosis and other conditions, and individualized pharmacotherapy considerations. Early multidisciplinary intervention, capacity assessments, and caregiver support are critical.

**Summary**

• Persistent delirium in elderly patients is frequently multifactorial, as demonstrated in this case with metabolic disturbances, polypharmacy, alcohol use, and underlying neurological issues all contributing to risk.
• This case underscores the need for vigilant monitoring, carefully considered diagnosis and pharmacotherapy, structured capacity assessment, and education for the surrogate decision‐maker, as well as robust multidisciplinary team involvement and support to optimize outcomes in this vulnerable population.

## 1. Introduction

Delirium is an acute, fluctuating disturbance of attention, awareness, and cognition, commonly affecting older adults, especially those with medical comorbidities and exposure to acute medical stressors [1–4]. While delirium is typically described as a transient syndrome, persistent delirium (defined as symptoms lasting weeks to months) has been increasingly recognized, with the rate of persistence varying by study and length of follow‐up, but with an estimated 27% at 12 months posthospital discharge [5–7]. Persistent delirium is associated with many negative effects, including higher rates of functional decline, institutionalization, and mortality, and is often under‐recognized due to its fluctuating nature and overlap with dementia [1, 4, 5, 7]. This is further complicated because those who develop delirium have higher odds of persistent cognitive decline and the development of dementia [5, 7, 8]. These findings highlight the importance of not only delirium prevention but also posthospital discharge follow‐up and tailored interventions for this vulnerable population [5–7].

The etiology of delirium is often multifactorial, with predisposing factors such as advanced age, prior neurological injury, polypharmacy, and substance use, and precipitating factors including infections, metabolic disturbances (notably hyponatremia), and medication effects [1, 2, 4, 9]. Alcohol use is a critical risk factor, as chronic consumption increases vulnerability through direct neurotoxicity, nutritional deficiencies, and exacerbation of comorbidities such as liver disease and electrolyte disturbances [2, 3, 10].

This case report illustrates an exceptionally prolonged case of persistent delirium with prominent paranoid delusions in a medically complex older adult, illustrating diagnostic and treatment challenges, and highlighting the potential role of mirtazapine when standard pharmacologic agents carry prohibitive risks.

## 2. Case Presentation

### 2.1. Patient Description

A 72‐year‐old Caucasian woman, retired educator with a master’s degree, with a history of left hemispheric hemorrhagic stroke (in 2003) resulting in right‐sided deficits and mild chronic expressive aphasia, poststroke epilepsy managed with divalproex sodium, hypertension, hyperlipidemia, and alcohol use disorder (averaging three glasses of wine daily for decades, in remission following delirium onset).

### 2.2. Premorbid Functioning

Prior to delirium onset, the patient lived with her husband, was independent with activities of daily living (limited only by wheelchair use), managed her own medications, and engaged in complex tasks and conversations. There was no history of progressive cognitive decline or dementia. The patient’s prior stroke caused mild expressive aphasia, yet with long‐term speech therapy the patient had regained fluent language abilities but reported occasional subjective expressive difficulties despite no impairment in her speech, and this was classified as mild chronic expressive aphasia by her neurology team.

### 2.3. Family and Psychiatric History

Family psychiatric history was notable only for an unknown tic disorder in her brother. The patient had no pertinent past psychiatric history other than a brief depressive episode during acute rehabilitation following her stroke, treated briefly with sertraline. There was no history of substance use other than alcohol.

### 2.4. Initial Presentation and Early Disease Course (Weeks 1–5)

Approximately 6 weeks before the patient presented to our institution, the patient experienced sudden confusion and speech difficulty while at dinner (see Figure 1). She reported feeling “strange” and was taken to the emergency department but left before full evaluation and, while there was refusing treatment and workup due to paranoia. Over the following weeks, the patient experienced waxing and waning confusion, agitation, and paranoid delusions, particularly persecutory beliefs that her husband and healthcare providers intended to harm her. Around 1 week prior to presenting to our institution, she left home alone at night and was found by police, disoriented in her wheelchair. The patient at baseline was able to locomote herself in her wheelchair and so did this to leave the home, albeit in a confused state. Police were able to contact the patient’s husband, who then took the patient to an emergency room. She was admitted to an outside hospital after being found to have low levels of sodium, potassium, and magnesium, and received corrective IV treatment. Despite improvements in her electrolyte levels, mental status fluctuated and did not consistently return to baseline.

**Figure 1:**
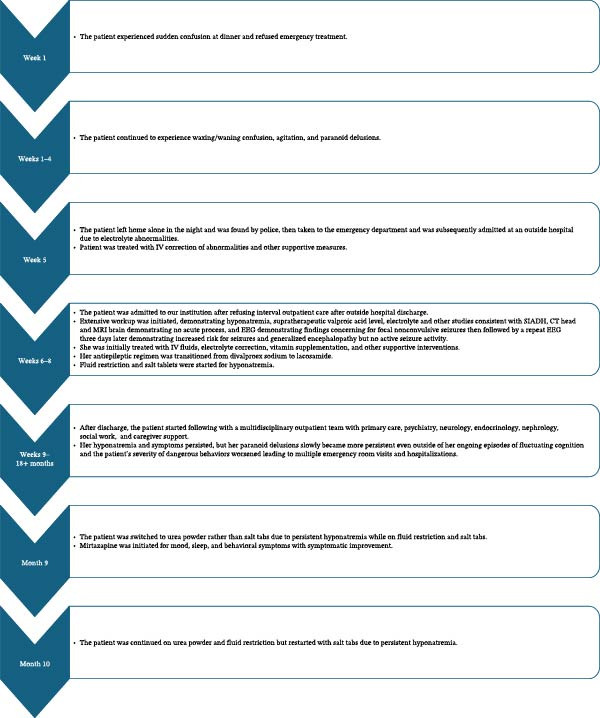
Temporal graph of the course of illness.

### 2.5. Intermediate Disease Course and Repeat Hospitalization (Weeks 6–8)

After discharge from the outside hospital, the patient refused outpatient care and symptoms persisted. Approximately 1 week after discharge from the outside hospital, the patient’s husband brought the patient to our institution due to the ongoing symptoms, where extensive workup and treatment were initiated.

### 2.6. Diagnostic Workup

Laboratory evaluation revealed persistently low sodium levels (122–134 mmol/L) despite fluid restriction, salt tablets, and later urea. Divalproex sodium was found to be supratherapeutic (up to 127 mcg/mL) in the setting of erratic medication adherence. SIADH was diagnosed as the etiology of her hyponatremia in the setting of euvolemic hypotonic hyponatremia and laboratory findings demonstrating urine sodium 118 mmol/L, urine osmolality 390 mOsm/kg, serum osmolality 275 mOsm/kg, and the exclusion of mimicking conditions including secondary adrenal insufficiency and hypothyroidism [11]. Laboratory workup included COVID‐19 testing, urinalysis, drug screening, TSH/T4, ammonia, B12, folate, thiamin, CBC, CMP, magnesium, creatinine kinase, phosphate, coagulation studies, iron studies, metanephrines and normetanephrines, serum protein electrophoresis, cortisol, aldosterone, renin, ACTH, DHEA‐sulfate, copeptin, LDH, alkaline phosphatase, and hepatitis panel and antibody testing showed no other significant abnormality that would be contributory to her presenting symptoms (see Table 1).

**Table 1:** Summary of diagnostic workup and results.

Test category	Specific test	Result
Electrolytes metabolic	Serum sodium	122‐134 mmol/L (persistently low)
	Serum osmolality	275 mOsm/kg
	Urine sodium	118 mmol/L
	Urine osmolality	390 mOsm/kg
	Magnesium	2.0 mg/dL
	Phosphate	3.4 mg/dL
Medication levels	Divalproex sodium	127 mcg/mL
Endocrine	TSH	3.318 uIU/mL
	T4	1.06 ng/dL
	Cortisol (baseline)	20.7 mcg/dL
	Cortisol (stimulation test)	27 > 26 at 30 and 60 min poststimulation
	ACTH	38 pg/mL
	Aldosterone	15 ng/dL
	Renin	1.49 ng/mL/h
	DHEA‐sulfate	37 mcg/dL
	Copeptin	3.8 pmol/L
Nutritional	Thiamin	116 nmol/L
	Vitamin B12	1376 pg/mL (high)
	Folate	24 ng/mL
Hematologic	CBC	No significant abnormality
	Coagulation studies	Normal
	Iron studies	No significant abnormality
	Serum protein electrophoresis	No significant abnormality
Hepatic	Ammonia	16 uMol/L
	Hepatitis panel and antibodies	Negative
Enzyme studies	Creatinine kinase	102 IU/L
	LDH	207 IU/L (high)
	Alkaline phosphatase	22 IU/L (low)
Infectious	COVID‐19 testing	Negative
	Urinalysis	No significant abnormality
Toxicology	Drug screening	Negative
Tumor markers	Metanephrines and normetanephrines	44 pg/mL and 54 pg/mL
Neuroimaging	CT head	No acute process
	Brain MRI	No acute process
	Chest/abdominal imaging	Incidental findings of 6 cm piriformis hematoma and 2 cm adrenal adenoma
Electrophysiology	Initial EEG	3–4 Hz rhythmic activity, intermixed sharp waves, focal slowing, generalized encephalopathy
	Repeat EEG (3 days later)	Epileptiform discharges, periodic discharges, generalized encephalopathy

Initial EEG demonstrated waxing and waning 3–4 Hz rhythmic activity in the left temporal region with intermixed sharp waves spreading to the left frontocentral region (concerning for focal nonconvulsive seizures), focal slowing in the left frontotemporal region (indicating underlying neuronal dysfunction), and mild generalized encephalopathy. A repeat EEG done 3 days later showed frequent epileptiform discharges in the left temporal and temporoparietal regions with periodic and lateralized periodic discharges, indicative of increased seizure risk and suggestive of recent recurrent seizures or underlying structural lesion but no active seizures, along with a mild degree of generalized encephalopathy.

A CT head demonstrated no acute infarction, mass, bleed, or fracture; a prior infarct‐related defect and encephalomalacia of the left insular lobe, basal ganglia, external capsule, and left thalamus; generalized mild‐to‐moderate cerebral atrophy most prominent in the left hemisphere; and patchy periventricular and subcortical white matter hypoattenuation consistent with chronic microvascular ischemic changes. This was interpreted by the radiologist to be an impression of “No acute intracranial abnormality is identified. Chronic microvascular ischemic disease. Generalized atrophy. Encephalomalacia in the left insula, consistent with prior infarct.” Brain MRI showed no acute infarction, no gross acute intracranial bleeding or mass, no sign of an acute inflammatory process, chronic nonspecific moderate diffuse atrophy, and nonspecific chronic degenerative changes favoring chronic hypervascular disease, which was interpreted by the radiologist to be an impression of “no gross acute intracranial process” (see Figure 2). Of note, the MRI was somewhat limited by motion artifact due to the patient’s limited ability to follow instructions (despite the patient receiving lorazepam prior to the MRI for temporary mild sedation to prevent this). Incidental findings from chest and abdominal X‐ray and CT scans included a 6 cm piriformis hematoma and a 2 cm left adrenal adenoma, neither acutely contributory to the patient’s symptoms. The patient denied any kind of associated pain, and all findings were negative for adrenal tumor overproduction in consultation with endocrinology; these were purely incidental findings.

**Figure 2:**
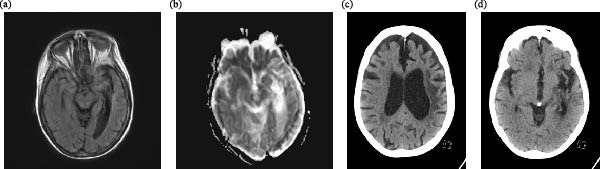
Neuroimaging findings. (a) Axial MRI (T2‐weighted/FLAIR) at the level of the hippocampi demonstrates chronic left temporal volume loss without medial temporal T2/FLAIR hyperintensity. Image quality is limited by motion artifact. (b) Axial diffusion‐weighted imaging shows no restricted diffusion. (c) Axial noncontrast CT at the level of the lateral ventricles demonstrates marked asymmetric cerebral atrophy with left hemispheric predominance and ex vacuo dilation of the left lateral ventricle. Periventricular white matter hypoattenuation consistent with chronic microvascular ischemic changes is also present. (d) Axial noncontrast CT at the level of the basal ganglia reveals chronic encephalomalacia involving the left insular cortex, lentiform nucleus, external capsule, and thalamus, consistent with sequelae of prior left middle cerebral artery territory hemorrhagic stroke.

### 2.7. Treatment Course

She was initially treated at our institution with intravenous fluids, electrolyte correction, vitamin supplementation (thiamin, folate, and multivitamin), and other supportive interventions, but mental status fluctuated and did not consistently return to baseline throughout admission. The antiepileptic regimen was transitioned from divalproex sodium to lacosamide (at initial admission to our institution) due to concerns for valproate‐induced encephalopathy and SIADH, in consultation with neurology. Hyponatremia management was done in consultation with nephrology (at initial admission to our institution) with salt tablets 1 g TID with meals and 1 L fluid restriction, but 7 months later, due to persistent hyponatremia, the patient was switched to urea powder 30 mg daily rather than salt tabs. Then, another month later, sodium tablets were added back to the regimen with urea due to ongoing hyponatremia. Mirtazapine was initiated (at ~9 months into the disease course) for mood, sleep, and behavioral symptoms with subsequent symptomatic improvement, selected for its lower hyponatremia risk compared to SSRIs/SNRIs, lower seizure risk compared to antipsychotics, and low deliriogenic potential (as discussed in detail below). Polypharmacy was minimized where possible.

### 2.8. Longitudinal Disease Course (9 Weeks to 18+ Months)

Despite some improvement in sodium levels and discontinuation of divalproex sodium, persistent delirium continued with ongoing paranoia, episodic agitation, and speech difficulties. There have been many additional emergency room visits and hospitalizations due to these persistent symptoms. Notably, her cognition returned to baseline between episodes, often rapidly enough that she would discharge home within hours of emergency department presentation. Over time, the clinical phenotype evolved: while cognition continued to return to baseline between episodes, paranoid delusions regarding medical professionals transitioned from episodic to nearly continuous. Even more dangerous behaviors emerged later in the disease course, including attempts to leave moving vehicles and an incident in which she attempted to stab her husband while he was driving her to a medical appointment, resulting in her first psychiatric hold and rehospitalization at our institution. The patient remains dependent on her husband during episodes due to the severity of her symptoms. Mirtazapine has been continued with sustained benefits for sleep and behavioral symptoms. She continues to follow up with a multidisciplinary team including primary care, psychiatry, neurology, endocrinology, nephrology, and social work, with caregiver support services engaged for ongoing management.

### 2.9. Barriers to Care

The patient’s paranoid delusions created significant treatment barriers. She frequently refused medications, laboratory monitoring, imaging studies, and follow‐up appointments. Early in the disease course, fluctuations in paranoia allowed intermittent treatment adherence. However, as paranoid delusions regarding medical professionals became more constant, nonadherence correspondingly became more constant. Her husband, serving as primary caregiver and surrogate decision‐maker, struggled to balance respecting the patient’s wishes while delusional with acting on treatment team recommendations.

## 3. Discussion

### 3.1. Persistent Delirium: Clinical Features and Outcomes

Delirium is classically defined as an acute, fluctuating syndrome of altered attention, awareness, and cognition, but persistent delirium has been increasingly recognized, especially in medically complex older adults [1, 6, 12]. Persistent delirium is associated with higher rates of functional decline, institutionalization, and mortality, and is often under‐recognized due to its fluctuating nature and significant clinical overlap with dementia [1, 4–7]. In this case, the patient’s delirium continues to persist despite medical interventions and continues to be characterized by waxing and waning confusion, persistent delusions, and behavioral disturbances.

### 3.2. Etiology: Multifactorial Contributors

The etiology of delirium is often multifactorial [1, 2, 4, 9]. In this patient, risk factors included advanced age, prior stroke, epilepsy, chronic hyponatremia (due to SIADH, poor oral intake, and possible effects from her thiazide and divalproex sodium use earlier in her disease course), polypharmacy, and significant alcohol use. Each of these factors is independently associated with increased risk of delirium [1–4, 9]. Alcohol use is a well‐established predisposing factor for delirium, even in the absence of withdrawal, and assessing for use is of particular importance in the geriatric populations, as discussed below [2, 3, 10, 13].

### 3.3. Alcohol Use and Neuropsychiatric Risk

The patient’s long‐standing alcohol use disorder (averaging three glasses of wine daily for decades) is a significant risk factor for both acute and persistent delirium, as well as for chronic neuropsychiatric conditions [10, 13]. Chronic alcohol use can exacerbate cognitive vulnerability, lower seizure threshold, and contribute to electrolyte disturbances, further increasing the risk of delirium and other neuropsychiatric issues [2, 10, 13]. Although the patient ceased alcohol use at the time of her initial hospitalization, the cumulative effects of decades of heavy use likely contributed to her presentation. Remembering to assess for alcohol and other substance use is essential because alcohol and other substance use is often overlooked and use disorder is often underdiagnosed in geriatric populations [14–18]. This is due to a multitude of factors, including the fact that the likelihood of primary care providers discussing alcohol use with patients declines as patient age increases, older adults may not recognize their substance use as problematic, screening tools validated for younger adults may be less sensitive in older populations, and others.

### 3.4. Polypharmacy and Drug Effects

Polypharmacy is a major risk factor for delirium in older adults, both through direct neuropsychiatric side effects and by increasing the risk of metabolic disturbances such as hyponatremia [1, 2, 4, 9]. In this patient, the combination of antihypertensives, antiepileptics, and other agents likely contributed to both the development and persistence of delirium. Notably, divalproex sodium was supratherapeutic and its neurotoxic effects may have contributed to the patient’s encephalopathy. Drug‐induced SIADH is also a recognized complication of several classes of medications which the patient was taking, including antiepileptics and thiazide diuretics [2]. Early evaluation of patient medication lists and optimization of medications to reduce polypharmacy as much as possible is of great importance, especially in cases such as this, when multiple medications are being used in combination that can have compounding side effects.

### 3.5. Diagnostic Complexity: Persistent Delirium vs. Other Differential Diagnoses

Distinguishing persistent delirium from major neurocognitive disorder and primary psychotic, mood, or other disorders is a frequent challenge in geriatric psychiatry, especially when these conditions may coexist and overlap [3, 4, 12]. In this case, the patient’s abrupt onset of symptoms with a waxing/waning course combined with her cognitive functioning returning to baseline between episodes argued against progressive dementia as her primary diagnosis. Additionally, the absence of any identifiable brain structural abnormality on CT head or MRI imaging that could explain her symptoms strongly argues against a structural cause. Wernicke encephalopathy diagnostic criteria were not met, as the patient did not have evidence of dietary deficiencies, eye signs, or cerebellar dysfunction, though the patient did have altered mental state. The clinical diagnosis requires at least two of those four findings, and furthermore, the patient had a normal thiamin level [19].

However, the inability to complete definitive diagnostic studies (repeat MRI, lumbar puncture, prolonged video‐EEG monitoring, etc.) due to patient nonadherence represents a major limitation to definitively differentiate between other possible diagnoses. Despite this, the clinical presentation is consistent with persistent delirium, rather than other potential differentials including ictal‐related psychosis, autoimmune encephalopathy, or a primary psychotic disorder (such as delusional disorder). The patient meets all criteria for delirium from the Diagnostic and Statistical Manual of Mental Disorders, fifth edition [20]. There is a clear disturbance in attention and awareness, the disturbance developed acutely and has fluctuated in severity over time, additional disturbances in cognition are present (cognitive domains of social cognition, executive function, and language), there was no evidence of preexisting dementia, the disturbances do not occur in the context of a severely reduced level of arousal or coma, and there is clear evidence of an underlying organic cause or causes (hyponatremia, supratherapeutic valproate levels, polypharmacy, alcohol use, etc.). Another supporting factor is that partial improvement was seen with metabolic correction when sodium was temporarily improved.

The frequent recurrence of her paranoia raised the possibility of ictal‐related psychosis (ictal, postictal, interictal) given the patient’s history of seizures, which is a well‐documented phenomenon that can occur in patients with epilepsy, particularly with temporal lobe involvement [21]. However, factors that support delirium rather than ictal‐related psychosis include that the temporal course of symptoms is highly characteristic of delirium in that the symptoms fluctuate rapidly throughout the day on a scale of hours and are of acute onset [22]. Ictal psychosis represents the briefest ictal phenomenon, lasting only seconds to minutes as a manifestation of the seizure itself, and is typically stereotyped and associated with automatisms or postictal confusion [23]. Although the EEG findings support delirium with underlying seizure risk, not ongoing ictal psychosis, as the EEG showed “waxing and waning 3–4 Hz rhythmic activity” and “frequent epileptiform discharges” concerning for focal nonconvulsive seizures initially but repeat EEG showed periodic discharges indicative of increased seizure risk rather than ongoing seizure activity, and symptoms were still present during this time. Furthermore, the patient’s time course of symptoms is inconsistent with postictal psychosis, as postictal psychosis has a mean duration of 9.2 days and resolves within 1 month in 95% of cases [24]. This patient’s symptoms have persisted for over 18 months since the initial presentation, which is far beyond the expected duration for postictal psychosis. There is an absence of the characteristic lucid interval, which postictal psychosis classically presents with a lucid interval of 12–72 h between seizure cessation and psychosis onset [25, 26]. Rather than the acute onset and fluctuating nature of delirium, postictal psychosis follows a more stereotyped pattern: it emerges after a relatively predictable lucid interval postseizure, reaches peak severity, then gradually resolves over days to weeks without the hour‐to‐hour fluctuation seen in delirium [24, 27]. Interictal psychosis represents a more chronic condition, with episodes lasting substantially longer [28]. The mean duration of interictal psychotic episodes is ~82.7 weeks; however, unlike the hour‐to‐hour fluctuations of delirium, interictal psychosis maintains relatively stable symptoms over extended periods, though it may eventually remit with treatment [28, 29]. Also, interictal psychosis typically presents with schizophreniform features including negative symptoms, which are absent in this case, and the acute onset temporally linked to metabolic derangements argues against interictal psychosis [30].

Autoimmune encephalopathy is a potential diagnosis but is unlikely in this case. The time of symptoms is more consistent with delirium than autoimmune encephalopathy, as symptoms progress over a period of days or weeks [31]. Notably absent were prodromal low‐grade fever, malaise, or headache, which occur in more than half of patients. Furthermore, some other prodromal symptoms that are characteristic of particular types of autoimmune encephalitides (faciobrachial dystonic seizures, paroxysmal dizzy spells, weight loss, etc.) were also absent. The diagnostic criteria for possible autoimmune encephalitis require both (1) subacute onset (<3 months) of working memory deficits, altered mental status, or psychiatric symptoms; and (2) at least one of the following: new focal CNS findings, seizures not explained by a pre‐existing seizure disorder, CSF pleocytosis, or MRI features of encephalitis (medial temporal T2/FLAIR hyperintensity or multifocal inflammatory changes) [32]. Regarding criteria two, the patient demonstrated no new focal CNS findings, no new seizures that were not explained by a pre‐existing seizure disorder, and no MRI features of encephalitis. Lumbar puncture could not be performed and so the criterion two cannot be completely excluded, though only 19% of patients with autoimmune encephalopathy have this finding. Due to the patient’s nonadherence to treatment recommendations it would be very difficult to complete a lumbar puncture and would require complete sedation of the patient. The high degree of inconsistency of this patient’s case with autoimmune encephalitis made it not favorable to perform a lumbar puncture when risks and benefits were weighed. Failure to fulfill both part 1 and 2 of the criteria precludes a diagnosis of any category of autoimmune encephalitis and therefore, although further work up (such as serum studies) could be of some benefit to differentiate between delirium vs autoimmune encephalopathy, it is of import to note that overinterpretation of positive serum antibodies is a demonstrated contributor to misdiagnosis of autoimmune encephalopathy [32].

A primary psychotic disorder, especially very late‐onset schizophrenia‐like psychosis (VLOSLP) and delusional disorder, is an important consideration in this case. However, many features make this diagnosis unlikely compared to delirium. There is a clear temporal relationship to medical precipitants in this case, whereas primary psychotic disorders typically have insidious onset without identifiable medical triggers [33, 34]. VLOSLP and other primary psychotic disorder symptoms are typically persistent and stable, not fluctuating, as in this case. The alteration in level of consciousness and rapid fluctuation of symptoms is a feature unique to delirium that is no characteristic of primary psychosis. The patient’s partial response to metabolic correction also favors delirium and would not be expected in a primary psychotic disorder. There is also an absence of characteristic VLOSLP or other primary psychotic disorder features, such as that VLOSLP typically presents with partition delusions and preserved cognitive function [35, 36].

### 3.6. Decision‐Making Capacity and Care Planning

Assessment of decision‐making capacity is critical in patients with persistent delirium [3]. This patient’s capacity was repeatedly assessed throughout her many medical encounters, yet there have been periods when she chose to refuse important medical treatment and workup despite documentation of her lack of capacity to make this decision. This highlights the need for regular, structured capacity assessments and clear communication with caregivers regarding surrogate decision‐making and their role as surrogate decision‐maker to make optimal choices for the patient [3].

### 3.7. Barriers to Treatment and the Importance of Early Intervention and Multidisciplinary Care

The patient’s paranoid delusions and intermittent refusal of care created significant barriers to effective treatment and follow‐up. Patients with delusions or other psychotic symptoms in the context of delirium are at risk for nonadherence to treatment recommendations due to conviction of the reality of their experience which leads to emotional and behavioral responses in line with their beliefs [1, 3, 4]. This case demonstrates the importance of early, proactive engagement with multidisciplinary teams (including social work, case management, caregiver support services, and medical specialty consultants) to address these barriers, prevent caregiver burnout, prevent escalation of behavioral issues, and ensure evaluation and treatment of all potentially contributing factors [3, 5]. Current literature supports early identification and management of precipitating factors for delirium, as well as specific education regarding substance use and its neuropsychiatric risks [1, 3, 5, 6, 10, 13]. In this case, earlier intervention may have mitigated some of the severe behavioral and psychosocial consequences experienced by the patient and her husband.

### 3.8. Role of Mirtazapine: Balancing Risk and Potential Benefit

Mirtazapine was associated with improvement in sleep, anxiety, and other behavioral/psychological symptoms in this patient, consistent with other studies demonstrating efficacy for its use in addressing sleep disturbance, depression, and other psychological symptoms (e.g., anxiety and pessimism) in older adults with chronic neuropsychiatric issues, specifically dementia [37, 38]. Although mirtazapine has not been studied specifically for persistent delirium, these findings are relevant considering the significant overlap of symptoms, pathogenesis, and other factors between persistent delirium and dementia, as already discussed above [1, 4, 5, 7, 8].

In this patient, mirtazapine was selected primarily for its superior safety profile compared to other potential pharmacologic treatment options for this patient’s symptoms. Mirtazapine has significantly lower risk of inducing or worsening hyponatremia compared to selective serotonin reuptake inhibitors, serotonin‐norepinephrine reuptake inhibitors, and tricyclic antidepressants [39–42]. Given the patient’s chronic severe hyponatremia (Na 122–134 mmol/L) and the contribution of hyponatremia to her delirium, avoiding medications that could further lower sodium was a critical consideration. Furthermore, mirtazapine has been shown to have a significantly lower risk of inducing or worsening seizures compared to antipsychotics, which are the standard pharmacologic treatment option for delirium but carry significant risk in this patient’s specific clinical circumstance [43–45]. Moreover, second‐generation antidepressants (other than bupropion), including mirtazapine, have demonstrated anticonvulsant effects [46–48]. Given the patient’s history of epilepsy and the potential contribution to her delirium and overall symptomatology, avoiding medications that could further worsen her seizures (antipsychotics) was another critical consideration. Additionally, mirtazapine has a very low deliriogenic risk, especially when compared to pharmacologic agents that are typically used as pharmacologic treatment of delirium (antipsychotics) [49].

While there is evidence supporting the potential for benefit with mirtazapine in treatment of persistent delirium in specific circumstances, such as this case, clinicians face a difficult choice. While mirtazapine carries low hyponatremia risk, potential anticonvulsant benefit, and very low deliriogenic risk, the lack of proven efficacy in patients with delirium as well as potential mortality signal and lack of benefit for agitation in patients with dementia must be weighed carefully [50]. In this case, the patient’s improvement in sleep, anxiety, and other behavioral/psychological symptoms may represent a sedative effect rather than treatment of underlying delirium. Nonpharmacologic interventions and treatment of underlying medical causes remain the cornerstone of delirium management [3]. However, when medications are indicated due to the severity of symptoms with significant impact on the patient’s quality of life and functioning (as in this case), mirtazapine may be a helpful option when other more typical pharmacologic agents carry significant risk outweighing potential benefit given the specific clinical circumstances, such as those described in this case. The risks and benefits must be carefully discussed with patients and surrogate decision‐makers, and close monitoring for adverse effects is essential.

### 3.9. Importance and Novelty

This case report makes multiple important contributions to the medical literature on persistent delirium in geriatric populations. First, it documents an exceptionally prolonged course of delirium lasting over 18 months, which extends beyond the typical duration reported in most studies of persistent delirium [6, 20]. To our knowledge, only one study has documented persistent delirium lasting up to 18 months, and in this study it was very rare [6, 51]. This case demonstrates symptoms persisting well beyond the typical timeframe for delirium, highlighting the potential for even more protracted courses in medically complex older adults.

Second, this case uniquely illustrates the prominent role of paranoid delusions as a presenting and persistent feature of persistent delirium. While psychotic symptoms occur in nearly 50% of delirium cases, delusions specifically are relatively uncommon, occurring in only 13%–14.6% of delirium patients [52, 53]. This case adds to the limited literature on delirium presenting with prominent and persistent paranoid delusions and especially the even more limited literature characterizing persistent delirium specifically. The evolution from episodic to continuous paranoid delusions over 18 months, despite partial improvement in precipitating factors (sodium correction, medication optimization), suggests that persistent delirium may undergo phenotypic changes over time, which is a phenomenon deserving further study.

Third, the case provides valuable insights into the diagnostic challenges of differentiating persistent delirium from other conditions in the context of multiple potential etiologies due to complex medical comorbidity. The patient presented with several conditions that could mimic the symptoms of or coexist with delirium. The detailed discussion of how to distinguish persistent delirium from ictal‐related psychosis, autoimmune encephalopathy, and primary psychotic disorders (particularly VLOSLP) contributes to the literature on differential diagnosis in complex geriatric cases.

Fourth, this case addresses the underrecognized role of chronic alcohol use disorder in geriatric delirium. This case emphasizes the importance of assessing alcohol use in geriatric populations and the consequences of chronic consumption.

Fifth, the case provides novel information on pharmacologic management considerations in the context of chronic hyponatremia and epilepsy. The decision to use mirtazapine was based on its significantly lower risk of inducing or worsening hyponatremia compared to other antidepressants, its lower seizure risk compared to antipsychotics, and its very low deliriogenic potential. This case illustrates a clinical scenario where the unique safety profile of mirtazapine may make it a helpful option for management when other pharmacologic agents carry prohibitive risks. The patient’s symptomatic improvements, though not representing resolution of delirium, demonstrates potential benefits in specific clinical contexts.

Finally, this case underscores the critical importance of multidisciplinary care, caregiver support, and early intervention (including capacity assessments and education of surrogate decision‐makers). The delay between initial symptoms and comprehensive evaluation and treatment may have allowed for more treatment difficulties, especially because the paranoid delusions were less prominent earlier on in the disease course and adherence was more likely at that time. The patient’s paranoid delusions created significant barriers to treatment adherence, highlighting the need for early, proactive engagement with multidisciplinary teams and caregiver support services to prevent escalation of behavioral issues and caregiver burnout. The challenges in obtaining informed consent from the patient and managing decision‐making capacity in the context of fluctuating cognition and persistent paranoia as well as the need for surrogate decision‐maker education offer important insights for clinicians managing similar cases.

## Funding

No funding was received for this work.

## Disclosure

The patient’s perspective could not be obtained due to ongoing symptoms that prevented meaningful conversation with the writer.

## Ethics Statement

This report was conducted in accordance with the Declaration of Helsinki. Institutional review board approval was not required per institutional policy for case reports. Written informed consent for publication was obtained from the patient’s husband, her healthcare proxy and legally authorized surrogate decision‐maker, as the patient herself lacked decisional capacity.

## Consent

The patient lacked decisional capacity throughout her care in our clinic system, as determined by serial capacity assessments performed at the time of initial evaluation and at subsequent follow‐up visits, and was unable to participate in discussion regarding consent for this report. Her husband, who serves as her designated healthcare proxy, was therefore approached as her legally authorized surrogate decision‐maker. After being provided with the consent form for publication, he reviewed it and provided informed consent on the patient’s behalf in a written email response, which was additionally verbally confirmed by telephone. A handwritten or electronic signature could not be obtained: he did not have access to electronic‐signature software on any of his devices, and he was unable to present to the clinic in person to sign a physical form because he and the patient had been displaced from their residence. His typed email statement of consent is retained on file in lieu of a signed form. All identifying information, including names, dates, and specific locations, has been removed or altered to protect the patient’s privacy and confidentiality.

## Conflicts of Interest

The author declares no conflicts of interest.

## Data Availability

Data sharing is not applicable to this article, as no datasets were generated or analyzed during the current study.
